# Differential responses of rumen and fecal fermentation and microbiota of Liaoning cashmere goats after 2-hydroxy-4-(methylthio) butanoic acid isopropyl ester supplementation

**DOI:** 10.1038/s41598-024-58581-y

**Published:** 2024-04-12

**Authors:** Zhiqiang Zhong, Peiyuan Sun, Yuning Zhang, Lingyun Li, Di Han, Xiaoguang Pan, Ruiyang Zhang

**Affiliations:** 1 College of Animal Science and Veterinary Medicine, Shenyang Agricultural University, Shenyang, 110866 China; 2 Liaoning Province Modern Agricultural Production Base and Construction Engineering Center, Shenyang, 110032 China; 3 School of Artificial Intelligence and Software, Liaoning Petrochemical University, Fushun, 113001 China; 4 State Key Laboratory of Swine and Poultry Breeding Industry, Guangzhou, China

**Keywords:** Rumen, Feces, HMBi, Fermentation, Microbial composition, Liaoning cashmere goats, Gastroenterology, Microbiology, Microbial communities

## Abstract

The 2-hydroxy-4-(methylthio) butanoic acid isopropyl ester (HMBi), a rumen protective methionine, has been extensively studied in dairy cows and beef cattle and has been shown to regulate gastrointestinal microbiota and improve production performance. However, knowledge of the application of HMBi on cashmere goats and the simultaneous study of rumen and hindgut microbiota is still limited. In this study, HMBi supplementation increased the concentration of total serum protein, the production of microbial protein in the rumen and feces, as well as butyrate production in the feces. The results of PCoA and PERMANOVA showed no significant difference between the rumen microbiota, but there was a dramatic difference between the fecal microbiota of the two groups of Cashmere goats after the HMBi supplementation. Specifically, in the rumen, HMBi significantly increased the relative abundance of some fiber-degrading bacteria (such as *Fibrobacter*) compared with the CON group. In the feces, as well as a similar effect as in the rumen (increasing the relative abundance of some fiber-degrading bacteria, such as *Lachnospiraceae FCS020 group* and ASV32), HMBi diets also increased the proliferation of butyrate-producing bacteria (including *Oscillospiraceae UCG-005* and *Christensenellaceae R-7 group*). Overall, these results demonstrated that HMBi could regulate the rumen and fecal microbial composition of Liaoning cashmere goats and benefit the host.

## Introduction

Methionine (Met) is an essential nutrient with multiple physiological functions that affect numerous aspects of ruminants, including lactation, growth and hair development^1–3^. Met can also serve as a methyl donor for cysteine (Cys), which is involved in protein translation and the synthesis of the antioxidant glutathione (GSH)^4^. However, the direct supplementation of Met has limited bioavailability, so strategies are required to ensure that it reaches the small intestine more efficiently for release. Consequently, rumen-protected methionine (RPMet) has been developed to resist rumen microbial degradation and to increase Met flow in the small intestine^5^.

The 2-hydroxy-4-(methylthio) butanoic acid isopropyl ester (HMBi), a type of RPMet, is widely used for this purpose. It is partially (more than 50%) degraded in the rumen and also serves as a source of Met for rumen microbiota^6,7^. Research has demonstrated that HMBi is rapidly absorbed through the rumen wall and metabolized into 2-hydroxy-4-(methylthio) butyric acid (HMB) and isopropyl ester^8^. The HMB is then transported through circulation to the liver and other organs, while the unabsorbed portion enters the small intestine and is passively diffused via the intestinal mucosa. In the liver and kidneys, HMB is converted to Met by D-2-hydroxy acid dehydrogenase, which is present in all tissue cells throughout the organism^9^. However, studies on HMBi have primarily focused on its effects on specific performance aspects in dairy cows, including milk yield and nitrogen metabolism. Relatively limited research has been conducted on Liaoning cashmere goats, specifically on the effects of HMBi on the hindgut^10^.

The degradation of nutrients by the gastrointestinal microbiota is pivotal in ruminants and determines their ability to utilize substances that are not available to monogastric animals^11^. Given this importance, and the ability of ruminants to break down complex nutrients, it is necessary to examine the effects of HMBi on the rumen and hindgut of ruminants to further our understanding of its impact on growth performance and other related aspects. Therefore, we hypothesize that HMBi supplementation may alter the composition of gastrointestinal microbiota, and the rumen and hindgut microbiota may respond differently to HMBi supplementation. Based on the above hypothesis, we conducted this study to explore the effects of HMBi on the serum biochemical parameters, rumen and fecal fermentation, and composition of microbiota in Liaoning cashmere goats to provide valuable knowledge about and better understand the benefits of HMBi in ruminants.

## Results

### Growth performance and serum biochemical indicators

There were no significant differences (*P* > 0.05) observed in BW and ADG between the CON group and HMBi group, while there is a reduced trend (*P* = 0.060) of F/G ratio in the HMBi group compared with the CON group (Table S2).

Compared with the CON group, the concentration of total serum protein was significantly increased (*P* = 0.026) in the HMBi group. However, no significant differences (*P* > 0.05) were observed between these two groups in terms of glucose, triglyceride, albumin, globulin, or urea nitrogen (Table S3).

### Rumen and fecal fermentation parameters

Compared with the CON group, the concentrations of rumen MCP were significantly increased (*P* = 0.038) in the HMBi group. However, there were no significant differences (*P* > 0.05) in rumen pH, acetate, propionate, butyrate, isobutyrate, valerate, isovalerate, or total volatile fatty acids (TVFAs) between the two groups (Table 1).

**Table 1:** Effects of 2-hydroxy-4-(methylthio) butanoic acid isopropyl ester (HMBi) supplementation on the fermentation parameters in the rumen and feces of Liaoning cashmere goats.

Items	Group		SEM	*P* value
	CON	HMBi		
Rumen				
pH	7.06	7.01	0.025	0.365
Acetate, mmol/L	31.84	31.00	1.574	0.798
Propionate, mmol/L	6.60	6.83	0.523	0.837
Butyrate, mmol/L	7.07	5.71	0.458	0.141
Isobutyrate, mmol/L	1.12	1.10	0.035	0.811
Valerate, mmol/L	0.49	0.53	0.038	0.621
Isovalerate, mmol/L	1.32	1.35	0.053	0.792
Total VFAs, mmol/L	48.44	46.51	2.439	0.706
MCP, mg/dL	24.03	28.62	1.135	0.038
Feces				
pH	8.02	8.41	0.117	0.117
Acetate, mmol/L	23.41	24.73	1.388	0.661
Propionate, mmol/L	3.17	3.89	0.247	0.267
Butyrate, mmol/L	1.47	2.20	0.170	0.026
Isobutyrate, mmol/L	0.50	0.49	0.026	0.891
Valerate, mmol/L	1.00	1.25	0.179	0.521
Isovalerate, mmol/L	0.40	0.41	0.028	0.873
Total VFAs, mmol/L	29.95	32.79	1.907	0.446
MCP, mg/dL	10.21	20.17	1.934	0.015

In feces, compared with the CON diet, the HMBi diet significantly increased the concentrations of butyrate (*P* = 0.026) and MCP (*P* = 0.015). However, no significant differences (*P* > 0.05) were observed between the two groups in terms of pH, acetate, propionate, isobutyrate, valerate, isovalerate, or TVFAs (Table 1).

### Microbial composition in the rumen and feces

**Figure 1:**
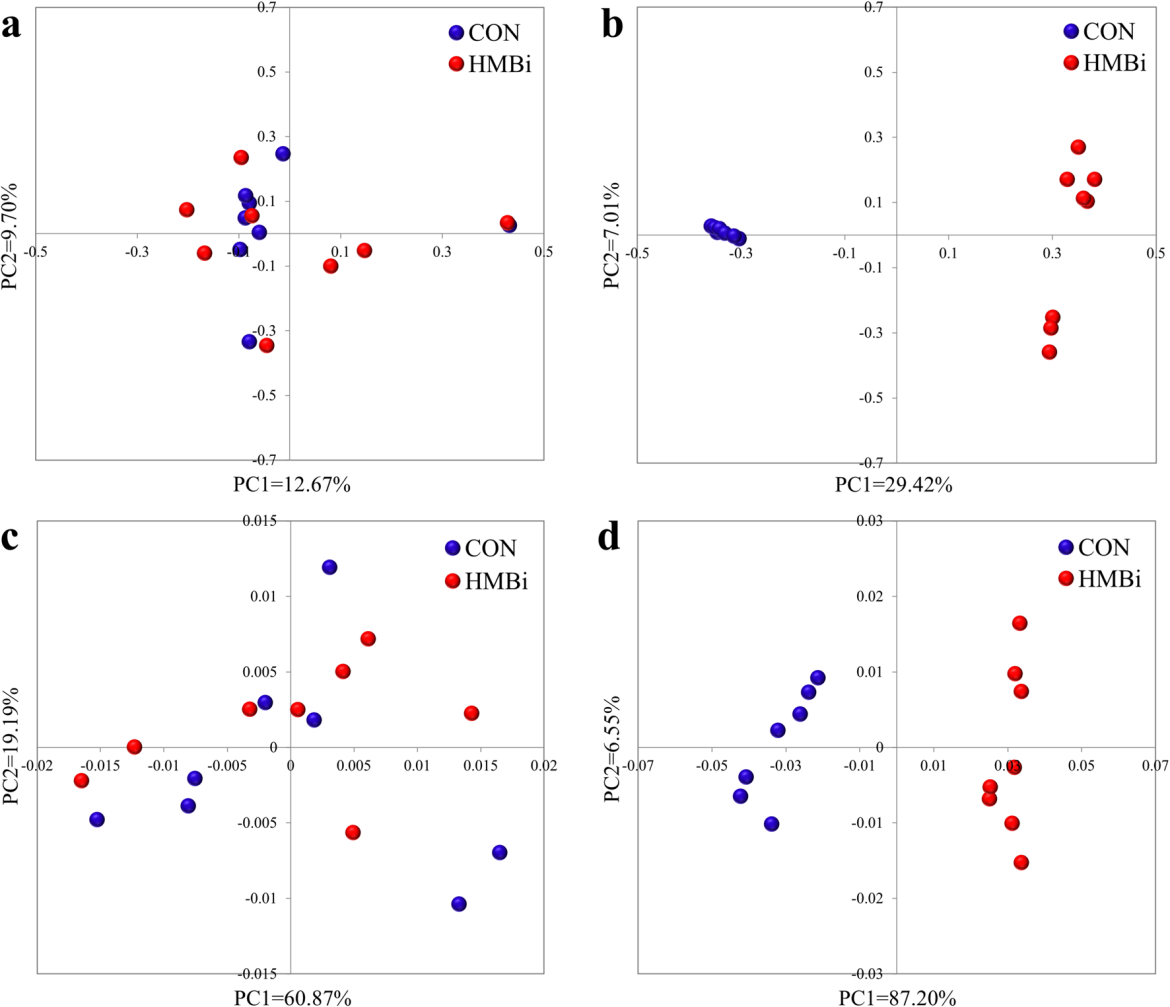
The principal coordinate analysis (PCoA) score plots of the microbial samples from the rumen (**a**) and feces (**b**) and PCoA score plots of the metabolic pathways (KEGG level 3) from the rumen (**c**) and fecal (**d**) microbiota, based on the Bray–Curtis similarity metric. CON, the control group; HMBi, the 2-hydroxy-4-(methylthio) butanoic acid isopropyl ester supplementation group.

**Figure 2:**
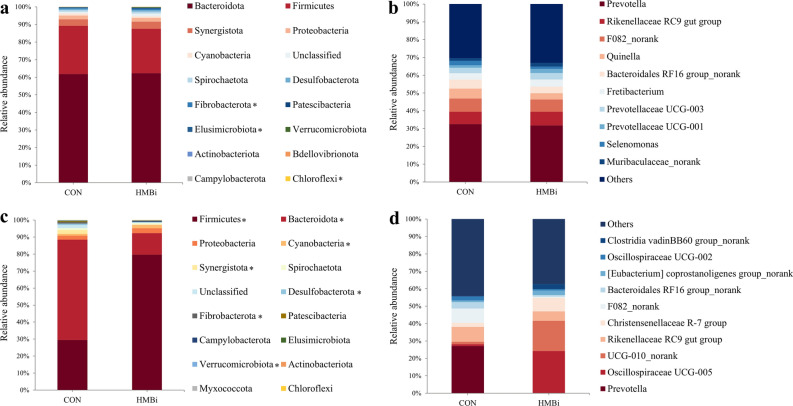
The microbial structure of the rumen and feces in Liaoning cashmere goat fed CON and HMBi diets at different taxonomic levels. The composition of the rumen microbiota at the phylum (**a**) and genus (**b**) levels. The composition of the fecal microbiota at the phylum (**c**) and genus (**d**) levels. CON, the control group; HMBi, the 2-hydroxy-4-(methylthio) butanoic acid isopropyl ester supplementation group. *P < 0.05.

**Figure 3:**
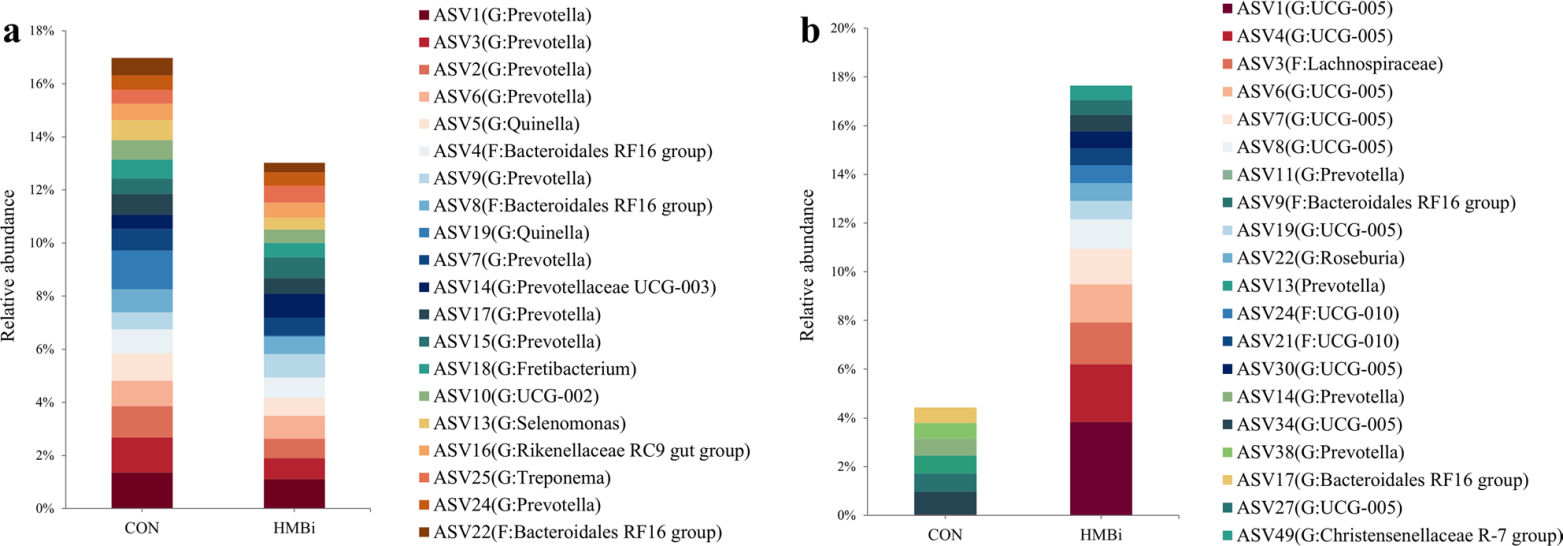
The microbial structure of the rumen (**a**) and feces (**b**) in Liaoning cashmere goat fed CON and HMBi diets at the ASV level. CON, the control group; HMBi, the 2-hydroxy-4-(methylthio) butanoic acid isopropyl ester supplementation group.

Regarding the ruminal microbiota, the results of PCoA and PERMANOVA (Fig. 1a) showed no significant differences between the CON and HMBi groups (PERMANOVA *P* = 0.659). At the phylum level, the predominant phyla were Bacteroidota, Firmicutes, Synergistota, Proteobacteria, and Cyanobacteria (Fig. 2a). At the genus level, the dominant genera were *Prevotella*, *Rikenellaceae RC9 gut group*, *F082_norank*, *Quinella*, and *Bacteroidales RF16 group_ norank* (Fig. 2b). At the ASV level (Fig. 3a), the dominant ASVs mainly included nine belonging to *Prevotella*, two belonging to *Quinella*, three belonging to *Bacteroidales RF16 group.*

The PCoA and PERMANOVA results (Fig. 1b) of the fecal microbiota indicated a significant difference (PERMANOVA *P* = 0.001) between the HMBi and CON groups. At the phylum level, the dominant phyla were mainly Firmicutes, Bacteroidota, Proteobacteria, Cyanobacteria, and Synergistota (Fig. 2c). At the genus level, the dominant genera were *Prevotella*, *Oscillospiraceae UCG-005*, *UCG-010_norank*, *Rikenellaceae RC9 gut group*, and *Christensenellaceae R-7 group* (Fig. 2d). At the ASV level, nine ASVs belonging to *UCG-005*, four belonging to *Prevotella*, two belonging to *UCG-010*, and two belonging to *Bacteroidales RF16 group*, were the predominant ASVs in the fecal microbiota (Fig. 3b).

### Microbial richness and diversity in the rumen and feces

For the microbiota in the rumen, α-diversity analysis showed that the Chao1 and Shannon indices were significantly higher (*P* < 0.05) in the HMBi group than in the CON group, while dietary treatment had no significant (*P* > 0.05) effect on the Simpson index (Table 2). For the fecal microbiota, as shown in Table 2, the Simpson index in the HMBi group was significantly lower (*P* < 0.05) than in the CON group, while dietary treatment had no significant (*P* > 0.05) effects on the Chao1 or Shannon indices.

**Table 2:** Effects of 2-hydroxy-4-(methylthio) butanoic acid isopropyl ester (HMBi) supplementation on the microbial diversity and richness of rumen and feces in Liaoning cashmere goat.

Items	Group		SEM	*P* value
	CON	HMBi		
Rumen				
Chao1	505	810	54	0.005
Shannon	5.58	5.95	0.065	0.001
Simpson	0.99	1.00	0.001	0.142
Feces				
Chao1	974	1060	107	0.704
Shannon	6.21	5.93	0.114	0.254
Simpson	1.00	0.99	0.001	0.042

### Alterations in the microbial composition of the rumen

**Figure 4:**
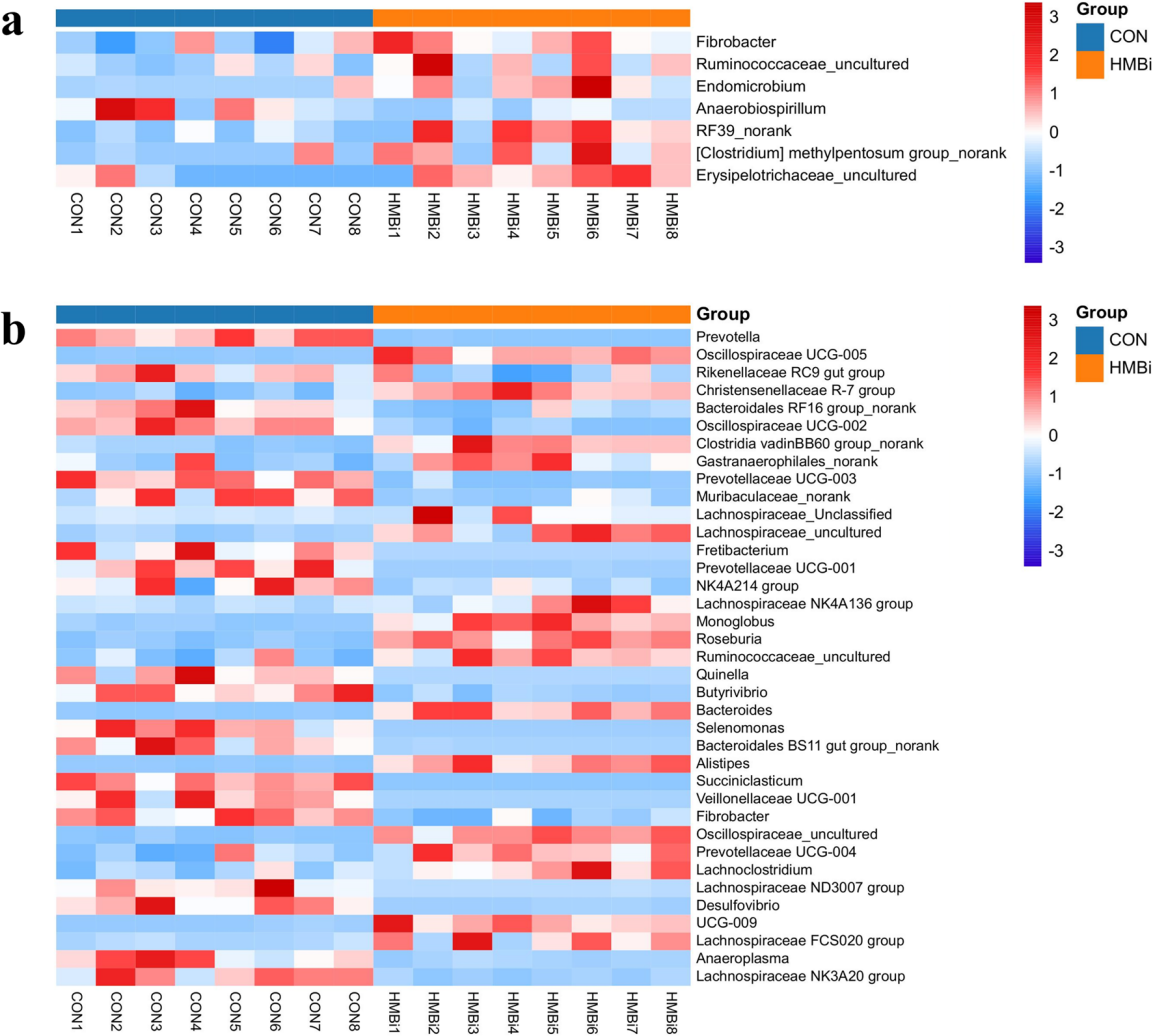
Statistical analysis of rumen (**a**) and fecal (**b**) microbiota at the genus level in Liaoning cashmere goat fed the CON and HMBi diets. CON, the control group; HMBi, the 2-hydroxy-4-(methylthio) butanoic acid isopropyl ester supplementation group. The heatmap plot was generated using R software (v.4.2.2) package “pheatmap” (v.1.0.12) through Hiplot Pro (https://hiplot.com.cn/).

At the phylum level, statistical analysis showed (Fig. 2a) that there were only minor differences in the ruminal microbiota following dietary treatment. The HMBi supplementation significantly increased (*P* < 0.05) the relative abundance of Fibrobacterota, Elusimicrobiota, and Chloroflexi compared to the CON group, while there was no significant (*P* > 0.05) effect on the other phyla. At the genus level, only seven genera were significantly affected by diet treatments (Fig. 4a). Compared with the CON group, the HMBi supplementation significantly increased (*P* < 0.05) the relative abundance of *Fibrobacter*, *Ruminococcaceae_uncultured*, *Endomicrobium*, *RF39_norank*, *[Clostridium] methylpentosum group_norank*, and *Erysipelotrichaceae_uncultured*, while that of *Anaerobiospirillum* was significantly reduced (*P* < 0.05), with no significant differences (*P* > 0.05) in the other genera.

**Figure 5:**
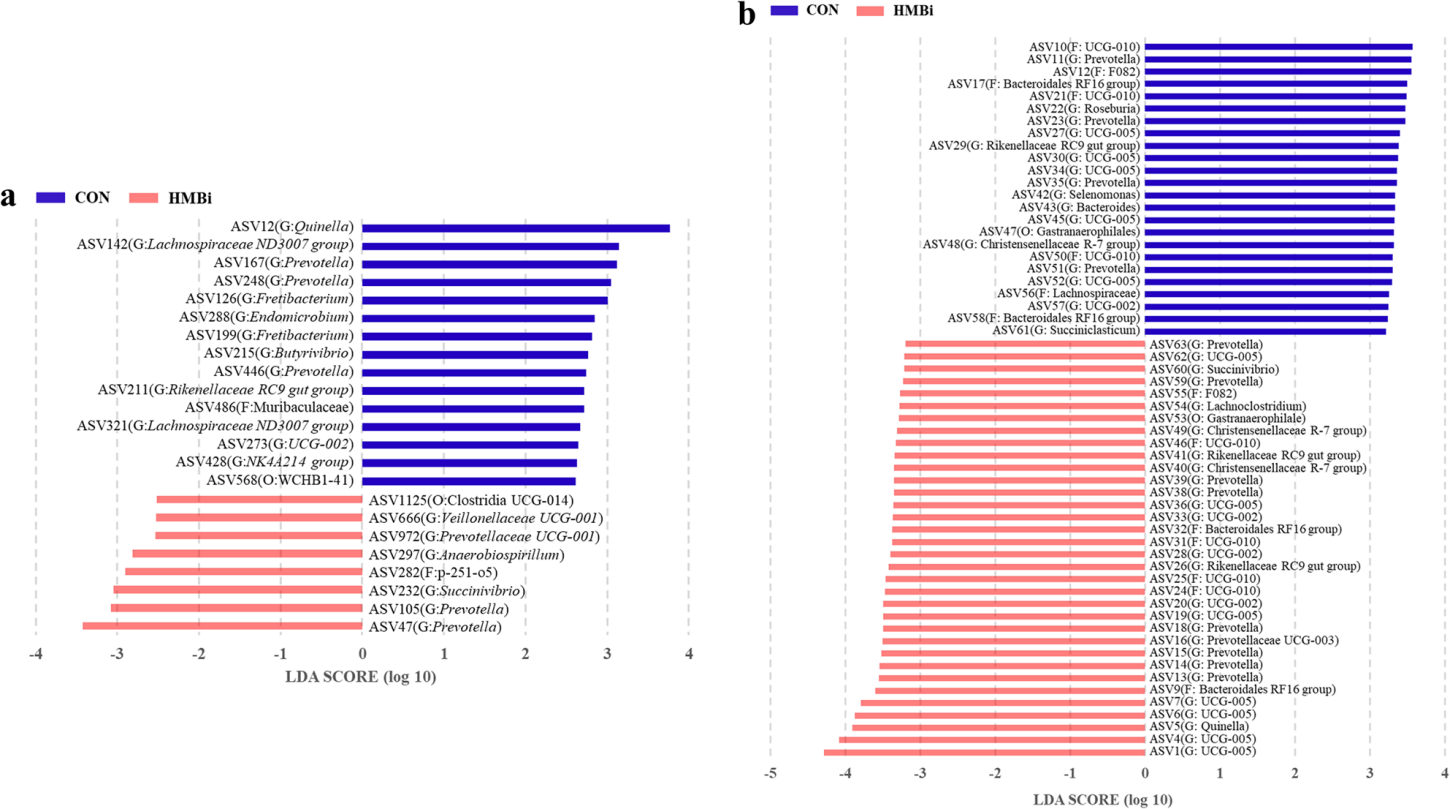
Linear discriminant analysis (LDA) scores of the microbial ASVs in the rumen (**a**) and feces (**b**) of Liaoning cashmere goat fed the CON and HMBi diets. CON, the control group; HMBi, the 2-hydroxy-4-(methylthio) butanoic acid isopropyl ester supplementation group.

In addition, LEfSe analysis was conducted at the ASV level to identify the characteristic microbiota in the rumen. The results showed that 23 ASVs were significantly (*P* < 0.05) affected by the goats’ diet, with 8 significantly (*P* < 0.05) enriched in the HMBi group and the remaining 15 significantly (*P* < 0.05) enriched in the CON group (Fig. 5a). Specifically, two ASVs belonging to *Prevotella*, ASV232 (G: *Succinivibrio*), ASV297 (G: *Anaerobiospirillum*), ASV972 (G: *Prevotellaceae UCG-001*), and ASV1125 (O: Clostridia UCG-014) were characteristic in the HMBi group (*P* < 0.05), while three ASVs belonging to *Prevotella*, two belonging to *Fretibacterium*, two belonging to *Lachnospiraceae ND3007 group*, ASV215 (G: *Butyrivibrio*), and ASV211 (G: *Rikenellaceae RC9 gut group*) were characteristic in the CON group (*P* < 0.05).

### Alterations in the microbial composition of the feces

At the phylum level (Fig. 2c), compared with the CON group, HMBi supplementation significantly increased (*P* < 0.05) the relative abundance of Firmicutes and Cyanobacteria, while significantly decreased (*P* < 0.05) the relative abundance of Bacteroidota, Synergistota, Desulfobacterota, Fibrobacterota and Verrucomicrobiota in the feces microbiota. At the genus level (Fig. 4b), compared to the CON group, the HMBi supplementation significantly increased (*P* < 0.05) the relative abundance of *Oscillospiraceae*, *UCG-005*, *Christensenellaceae R-7 group*, *Lachnospiraceae NK4A136 group*, *Monoglobus*, *Roseburia*, *Bacteroides*, *Alistipes*, *Prevotellaceae UCG-004*, and *Lachnospiraceae FCS020 group*, while significantly decreasing (*P* < 0.05) the relative abundance of *Prevotella*, *Rikenellaceae RC9 gut group*, *Oscillospiraceae UCG-002*, *Prevotellaceae UCG-003*, *Fretibacterium*, *Prevotellaceae UCG-001*, *Quinella*, *Butyrivibrio*, *Selenomonas*, and *Fibrobacter*.

At the ASV level, the results of LEfSe analysis showed that a total of 58 ASVs were significantly (*P* < 0.05) affected by the diet, of which 24 and 34 were significantly enriched in the CON and HMBi groups, respectively (Fig. 5b). More specifically, four ASVs belonging to *Prevotella*, five belonging to *UCG-005*, two belonging to *Bacteroidales RF16 group*, ASV29 (G: *Rikenellaceae RC9 gut group*), ASV42 (G: *Selenomonas*), ASV56 (F: Lachnospiraceae), and ASV61 (G: *Succiniclasticum*) were the characteristic microbiota in the feces of the CON group (*P* < 0.05), while eight ASVs belonging to *Prevotella*, seven belonging to *UCG-005*, two belonging to *Bacteroidales RF16 group*, two belonging to *Christensenellaceae R-7 group*, and two belonging to *Rikenellaceae RC9 gut group* featured in the HMBi group (*P* < 0.05).

### Metabolic pathway prediction of rumen and fecal microbiota

**Figure 6:**
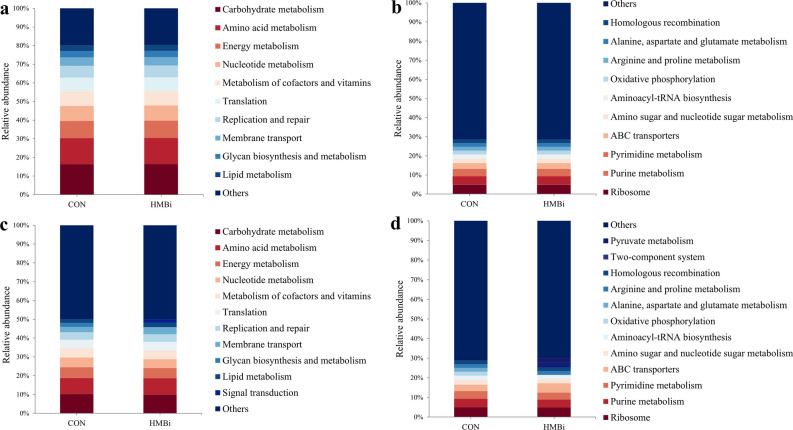
The predominant metabolic functions of rumen and fecal microbiota in Liaoning cashmere goat fed the CON and HMBi diets at different KEGG levels. The metabolic functions of the rumen microbiota at KEGG level 2 (**a**) and 3 (**b**). The metabolic functions of the fecal microbiota at KEGG level 2 (**c**) and 3 (**d**). CON, the control group; HMBi, the 2-hydroxy-4-(methylthio) butanoic acid isopropyl ester supplementation group.

Next, we predicted the potential function of the microbiota using PICRUSt. In the rumen, carbohydrate metabolism (average 16.36%), amino acid metabolism (average 14.03%), energy metabolism (average 9.24%), nucleotide metabolism (average 8.13%), and metabolism of cofactors and vitamins (average 7.69%) were the dominant pathways at KEGG level 2 (Fig. 6a). In terms of specific pathway differences between the two groups, the relative abundance of endocrine and metabolic disease was significantly reduced (*P* = 0.007) in the HMBi group compared to the CON group (Table 3). Among the 205 predicted metabolic pathways, at KEGG level 3, ribosome (average 5.00%), purine metabolism (average 4.31%), pyrimidine metabolism (average 3.82%), ABC transporters (average 3.05%), and amino sugar and nucleotide sugar metabolism (average 2.32%) were the dominant pathways (Fig. 6b). The results of PCoA and PERMANOVA showed no significant differences (PERMANOVA *P* = 0.72) between the two groups at KEGG level 3 (Fig. 1c), and LEfSe analysis also indicated that no characteristic metabolic pathways were enriched in either group (*P* > 0.05).

**Table 3:** The alterations in the metabolic functions of rumen and fecal microbiota at KEGG level 2 in Liaoning cashmere goat fed the control (CON) and 2-hydroxy-4-(methylthio) butanoic acid isopropyl ester (HMBi) diets.

Items	Group		SEM (%)	*P* value
	CON (%)	HMBi (%)		
Rumen				
Endocrine and metabolic disease	0.20	0.19	0.001	< 0.001
Feces				
Carbohydrate metabolism	16.38	15.64	0.107	0.005
Biosynthesis of other secondary metabolites	1.79	1.33	0.063	0.002
Xenobiotics biodegradation and metabolism	1.57	1.92	0.054	0.002
Energy metabolism	9.27	8.55	0.097	0.001
Lipid metabolism	3.15	3.54	0.052	0.001
Nucleotide metabolism	8.18	7.55	0.085	0.001
Metabolism of other amino acids	2.63	2.31	0.044	0.001
Glycan biosynthesis and metabolism	3.31	2.12	0.157	0.001
Metabolism of cofactors and vitamins	7.64	7.16	0.066	0.001
Metabolism of terpenoids and polyketides	2.06	2.18	0.017	< 0.001
Transcription	1.41	1.67	0.036	< 0.001
Membrane transport	4.64	6.09	0.201	< 0.001
Signal transduction	2.00	3.00	0.134	< 0.001
Transport and catabolism	0.55	0.32	0.031	< 0.001
Cell growth and death	1.13	1.00	0.017	< 0.001
Cell motility	1.49	2.74	0.166	< 0.001
Environmental adaptation	0.28	0.35	0.010	< 0.001
Endocrine system	0.67	0.60	0.009	< 0.001
Digestive system	0.15	0.03	0.016	< 0.001
Excretory system	0.02	0.05	0.005	< 0.001
Immune disease	0.09	0.05	0.005	< 0.001
Infectious disease: viral	0.00	0.00	0.000	< 0.001
Amino acid metabolism	13.68	13.90	0.036	< 0.001
Folding, sorting and degradation	2.79	2.87	0.013	0.009
Infectious disease: bacterial	0.18	0.17	0.002	0.008
Cancer: overview	0.12	0.11	0.002	0.023
Endocrine and metabolic disease	0.19	0.19	0.001	0.050

**Figure 7:**
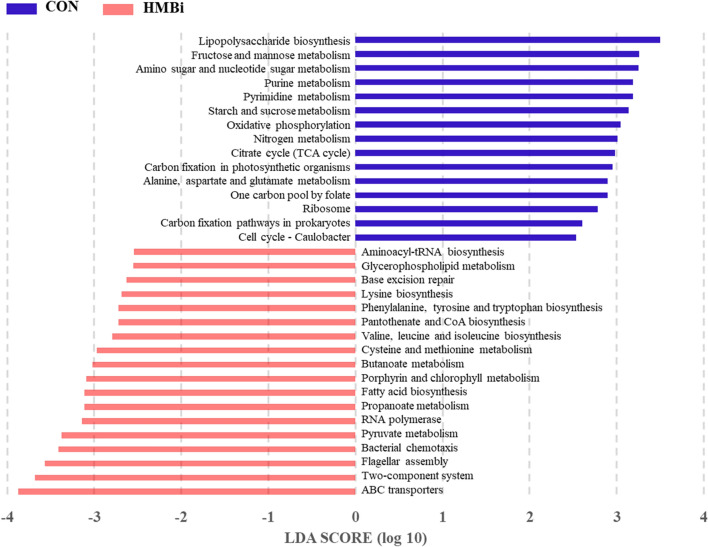
Linear discriminant analysis (LDA) scores of the microbial metabolic functions (KEGG level 3) in the feces of Liaoning cashmere goat fed the CON and HMBi diets. CON, the control group; HMBi, the 2-hydroxy-4-(methylthio) butanoic acid isopropyl ester supplementation group.

In the feces analysis, carbohydrate metabolism (average 16.01%), amino acid metabolism (average 13.79%), energy metabolism (average 8.91%), nucleotide metabolism (average 7.87%), and metabolism of cofactors and vitamins (average 7.40%) were dominant at KEGG 2 level (Fig. 6c). According to statistical analysis, the relative abundance of 11 pathways was significantly increased (*P* < 0.05) in the HMBi group compared to the CON group, including amino acid metabolism, lipid metabolism, metabolism of terpenoids and polyketides, xenobiotics biodegradation and metabolism, and membrane transport, whereas the relative abundance of 16 metabolic pathways was significantly decreased (*P* < 0.05), including those of energy metabolism, metabolism of other amino acids, glycan biosynthesis and metabolism, metabolism of cofactors and vitamins, and immune diseases (Table 3). At KEGG level 3, a total of 206 metabolic pathways were predicted, with ribosome (average 4.97%), purine metabolism (average 4.19%), pyrimidine metabolism (average 3.68%), ABC transporters (average 4.03%) and amino sugar and nucleotide sugar metabolism (average 2.17%) dominating, ignoring dietary treatments (Fig. 6d). The results of PCoA and PERMANOVA indicated significant differences (PERMANOVA *P* = 0.01) between the two groups at KEGG level 3 (Fig. 1d). In addition, the LEfSe analysis showed that 33 metabolic pathways were significantly (*P* < 0.05) impacted. More specifically, 18 pathways, including pyruvate metabolism, cysteine and methionine metabolism, lysine biosynthesis, valine, leucine and isoleucine biosynthesis, and butanoate metabolism, were significantly enriched (*P* < 0.05) in the HMBi group. The remaining 15 metabolic pathways were significantly enriched (*P* < 0.05) in the CON group, including the citrate cycle (TCA cycle), oxidative phosphorylation, nitrogen metabolism, lipopolysaccharide biosynthesis and starch and sucrose metabolism (Fig. 7).

## Discussion

In this study, the F/G ratio showed a decreasing trend after HMBi supplementation, while there were no significant differences in the BW and ADG between the CON and HMBi groups. The HMBi supplementation increased the amount of Met in the small intestine and balanced the proportion of amino acids, which improved the digestibility and absorption of amino acids and promoted protein synthesis. The increased concentration of total serum protein in this study confirmed this perspective. A previous study reported that no significant difference in BW, ADG, and F/G ratio of finishing beef cattle after feeding HMBi diets for 56 days, but significant differences at 105 days of feeding^6^. Therefore, we speculated that the results of this study were related to the shorter feeding cycle. The changes in the F/G ratio indicated that the HMBi supplementation may have a positive effect on growth performance.

Serum biochemical parameters are comprehensive indicators of the body’s metabolism and health status. In this study, the dietary supplementation of HMBi significantly increased the concentration of total serum protein in Liaoning cashmere goats. Consistent with our results, the Han et al. (2017) found that an HMBi supplementation dose of 15 g/d to the diet of Holstein cows significantly increased the concentration of total serum protein^8^. In the present study, the increase in total serum protein in the HMBi group probably reflected an increase in protein synthesis in the goats’ lives. However, no significant differences were observed between the two groups in glucose, triglyceride, albumin, globulin, or urea nitrogen. Overall, the above results indicate that supplementation of HMBi does not cause adverse effects on animal health.

In this study, HMBi supplementation had no significant effect on pH or VFA concentration in the rumen, but MCP concentration increased. Salter et al. (1979) found that Met was the first limited amino acid for rumen bacterial growth and that the provision of Met could enhance the growth and reproduction of rumen microbiota and promote the synthesis of rumen MCP^12^. Consistent with the rumen results, we found that the concentration of fecal MCP increased after HMBi supplementation. In addition, our results demonstrated that butyrate concentration was significantly higher in the HMBi group than in the CON group. Butyrate supplies energy and promotes the health of intestinal cells, hence HMBi supplementation would have a positive effect on hindgut health.

In general, the results of PCoA and PERMANOVA showed no significant difference in the richness and diversity of the ruminal microbiota, but there was a dramatic difference between the fecal microbiota of the two groups of Cashmere goats after HMBi supplementation. This result was similar to a previous report that found significant differences in the β-diversity of cecum but not ruminal microbial composition after the dietary supplementation with HMBi^6^. Another study demonstrated that approximately 50% of ingested HMBi was degraded to HMB and utilized by microbiota in the rumen to synthesize MCP^13^. These inconsistent microbial changes in the rumen and hindgut may therefore be related to differences in the reached substrates, but the exact mechanism still needs further study. Furthermore, we conducted microbial diversity analysis of the ruminal and fecal microbiota, and our findings show that HMBi increase microbial diversity in both the rumen (increased Chao 1 and Shannon indice) and feces (decreased Simpson index). Numerous studies have confirmed that relatively stable microbial abundance and diversity benefit the gastrointestinal microenvironment and the host’s health^14,15^. From this perspective, HMBi supplementation would help maintain the microbial diversity of the digestive tract, which in turn is beneficial to health status.

The microbiota of the rumen is extremely sophisticated and closely related to the physiological status and diet structure of the host^16,17^. Consistent with other studies, we found that Bacteroidota and Firmicute were the main dominant bacterial phyla, regardless of dietary treatment^18,19^. These dominant microbiota play a vital role in the degradation of ingested feed, especially basic substances such as proteins, cellulose, and starch, which are the primary energy source for the growth of the host and the microbiota^20,21^. As to specific differences, our results showed that the phylum Fibrobacteres and the genus *Fibrobacter* and *Ruminococcaceae_uncultured* were significantly higher in the HMBi group. *Fibrobacter* and Ruminococcaceae are the major cellulolytic bacteria in the rumen, and they were important in the degradation process of fiber. Similar to our results, Martin et al. (2013) reported that supplementating the diet of cattle with HMBi significantly increased the ruminal abundance of *Fibrobacter succinogenes* and *Ruminococcus flavefaciens*^22^. Additionally, previous studies have also confirmed that Met analogs are able to stimulate carboxymethylcellulase activity and rumen microbiota, including *Fibrobacter succinogenes* and *Ruminococcus flavefaciens*, to promote the production of MCP^22,23^. Hence, the increase in the abundance of these bacteria demonstrated that HMBi stimulated the proliferation of fiber-degrading bacteria, which was part of the reason for the increase in MCP.

In addition to fiber-degrading bacteria, our results showed that two ASVs belonging to *Prevotella* (ASV47 and ASV105), ASV232 (G: *Succinivibrio*), ASV297 (G: *Anaerobiospirillum*), ASV972 (G: *Prevotellaceae UCG-001*) and ASV1125 (O: Clostridia UCG-014) were enriched in the rumen of the HMBi group. *Prevotella*, a member of the Prevotellaceae family, is the dominant bacterial genus in the rumen, as shown by the bacterial analysis in the present study. *Prevotella* species have multiple metabolic functions and are involved in the degradation of various substrates, such as carbohydrates, fibers, and proteins^24,25^. An in vitro study showed that the abundance of *Prevotella* was significantly higher in the HMBi group than in the control group and that *Prevotella* was significantly positively correlated with dry matter digestibility, MCP, and fermentation parameters^26^. The genera *Succinivibrio* and *Anaerobiospirillum* are both members of the Succinivibrionaceae family, which is a common and important microbiota in the rumen. Previous studies have indicated that Succinivibrionaceae contribute to the degradation of starch, pectin, and dextrin, with fermentation products dominated by acetate and propionate (generating succinate, an intermediate in the production of propionic acid), and promote the formation of MCP^27,28^. In addition, Succinivibrionaceae members were reported to be positively correlated with feed efficiency in dairy cows and goats^29,30^. Overall, the above results suggested that the dietary supplementation of HMBi had a certain impact on the rumen bacteria involved in carbohydrate degradation, as well as affecting the fiber-degrading bacteria.

In addition to the rumen, hindgut fermentation is also very important to the ruminant host. For example, previous studies have shown that caecal fermentation could provide about 12% of the total VFAs of ruminants^31^. We therefore conducted microbial analysis of the feces in the present study. At the phylum level, and similarly to the ruminal microbiota, Firmicutes and Bacteroidota were the dominant phyla in the feces. We also observed an increase in the relative abundance of Firmicutes and a decrease in Bacteroidota in the HMBi group. As previously mentioned, the changes in these two important bacterial phyla indicate that HMBi has a huge impact on fecal microbiota. In addition, we also found a significant decrease in the relative abundance of Fibrobacterota after HMBi supplementation. A possible explanation for this decrease is that HMBi supplementation enhances fiber degradation in the rumen, leading to a decreased amount of fiber substrates reaching the hindgut. Desulfobacterota are sulfate-reducing bacteria whose members are able to reduce sulfate to hydrogen sulfide^32,33^. In this study, the decreased abundance of Desulfobacterota in the HMBi group indicated that the HMBi diet may help maintain host health and benefit the housing environment.

At a lower taxonomic level, we observed that the relative abundance of some butyrate-producing bacteria, such as *Christensenellaceae R-7 group* (including ASV40 and ASV49), *Lachnospiraceae* (NK4A136 group and FCS020 group), *Roseburia*, and *Alistipes*, was significantly higher in the HMBi group compared to the CON group. Except for *Alistipes*, these all belong to the phylum Firmicutes, which exhibits sugar-degradation activity that results in the production of butyrate, among others. In human intestinal studies, *Alistipes* has been shown to secrete butyrate^34^. These changes in butyrate-producing bacteria correspond to the significant increase in fecal butyrate concentration after HMBi supplementation in the present study. Numerous studies have confirmed that butyrate plays a vital role in cellular energy supply, immune regulation and inflammation elimination^35,36^. Consequently, these changes in the relative abundance of butyrate-producing bacteria and butyrate concentration may have a positive effect on the hindgut health and performance of goats after HMBi supplementation.

Our results also showed that *Prevotella* and *Butyrivibrio* were significantly decreased in the HMBi group compared to the CON group. As previously mentioned, *Prevotella* members have multiple metabolic functions and are involved in the degradation of many substances. The variation in *Prevotella* abundance could be substrate-dependent, presumably brought on by a greater rate of degradation and absorption of ingested material by the rumen and small intestine. Nevertheless, the significant reduction in *Butyrivibrio*, which belongs to the same Firmicutes family and utilizes butyrate as the primary metabolite, in the HMBi group, may be due to the competition for substrates between different genera or species. Because bacterial species rarely work together, competition is the dominant interaction^37^.

Our results showed that the functions of the microbiota in the rumen and feces were the same at KEGG level 2, mainly carbohydrate metabolism, amino acid metabolism, energy metabolism and nucleotide metabolism. These functions ensure the normal growth and proliferation of the microbiota resident in the digestive tract. Statistical and LEfSe analysis of the rumen showed that only one pathway (endocrine and metabolic disease metabolic) was affected by the HMBi diet, indicating that HMBi has a minimal effect on the metabolic pathways of the rumen microbiota, which corresponded with the limited changes observed in the microbiota itself.

Unlike these minimal changes in the rumen, we found dramatic differences in fecal microbial function after HMBi supplementation. Our results indicated that four pathways relating to amino acid metabolism (including cysteine and methionine metabolism, phenylalanine, tyrosine and tryptophan biosynthesis, lysine biosynthesis, and valine, leucine and isoleucine biosynthesis) feature in the HMBi group. HMBi supplementation increased hindgut Met flow, and Met acts as a methyl donor to generate Cys, leading to improved Cys and Met metabolism. Apart from this one, the other amino acid-related pathways significantly affected by the dietary treatment were all synthetic. This change could result in greater MCP synthesis, which was confirmed by the increased MCP concentration in the feces of the goats fed the HMBi diet. Moreover, our results showed that the pathway, butanoate metabolism, was enriched in the HMBi group, and this corresponded to the increased butyrate concentration and abundance of butyrate-producing microbiota in the HMBi group. Our results also showed that the abundance of some catabolic pathways (such as alanine, aspartate and glutamate metabolism, purine metabolism, and nitrogen metabolism) was reduced in the HMBi group, indicating that more nitrogen sources are utilized by the hindgut microbiota to synthesize microbial proteins after HMBi supplementation. Our prediction was supported by a previous study showing that the dietary supplementation of HMBi significantly reduces nitrogen excretion and improves nitrogen utilization in cows^13^. Additionally, our results indicated the lipopolysaccharide (LPS) biosynthesis pathway was relatively attenuated in the HMBi group compared to the CON group. In general, the release of LPS is caused by the massive disintegration of Gram-negative bacteria, and Bacteroidota was the most abundant Gram-negative bacteria found in the animal digestive tract^38^. Consequently, the decrease in the abundance of Bacteroidota and in the LPS biosynthesis pathway in the feces of goats fed the HMBi diet in the present study may lead to a decrease in fecal LPS concentration, and be of benefit to hindgut health.

In conclusion, our study found differences in serum biochemical indicators, rumen and fecal fermentation, and microbiota responses to an HMBi diet in Liaoning cashmere goats. Our results suggested that host protein nutrition was improved, and that rumen and fecal fermentation and microbiota changes were partially similar after treatment, but fecal microbial changes were more dramatic. In the rumen, HMBi supplementation increased some fiber-degrading bacteria (such as *Fibrobacter* and ASV232). In the feces, as well as a similar effect as in the rumen (increasing some fiber-degrading bacteria, such as *Lachnospiraceae FCS020 group*, ASV9, and ASV32), an HMBi diet also increased the proliferation of butyrate-producing bacteria (including *Oscillospiraceae UCG-005*, *Christensenellaceae R-7 group*, ASV40, and ASV49). Overall, these findings suggested that HMBi supplementation has a certain positive impact on ruminal and fecal fermentation and on microbial composition and function. Further research is needed to understand the specific mechanisms responsible for these changes and to determine their potential implications for animal health and productivity.

## Methods

### Experimental animals and diets

The study is reported following the ARRIVE guidelines (https://arriveguidelines.org) and all protocols in this study were approved by the Institutional Animal Care and Use Committee of Shenyang Agricultural University, Shenyang, China (IACUC No. 2021091501). A total of 16 Liaoning cashmere goats with similarly original ages (8-month-old) and approximate body weight (39.94 ± 0.886 kg) have randomly been allotted to 2 groups (n = 8), which one feed control diet, and another feed experimental diet (basic diets + HMBi, supplied by Adisseo company, with 1.27% DM). The experimental period was 47 days: 7 d for adaptations and 40 d for testing. The comprehensive nutrition and composition of the basic diet are shown in Table S1.

The body weight (BW) of each Liaoning cashmere goat was measured on the first and last day of the experimental period before morning feeding, respectively. The average daily gain (ADG) was calculated as the difference between final BW and initial BW and divided by 40. The feed/gain (F/G) ratio was determined by dividing the average daily feed intake (the sum of the difference between the daily diet offered to and refused by goats, and then divided by the number of days of the experiments) by ADG. Jugular vein blood was collected in triplicate using EDTA tubes (5 ml) before the morning feeding on day 37 of the formal experimental period. Samples were centrifuged at 3500 × g for 15 min, and the serum was collected and stored at − 80 °C for subsequent processing. After the first feeding on day 40 of the formal experimental period (waiting for 4–5 h), the rumen content and feces were collected. Homogenization of the rumen content and feces was performed, and respective portions were gathered into sterile centrifuge tubes and immediately put aside a − 80 °C for subsequent DNA extraction.

### Fermentation and serum biological parameters determination

Rumen content and feces pH were measured using a portable pH meter (pH400, Alalis Instrument, Inc., Shanghai, China). The volatile fatty acids (VFA) were measured using high-performance gas chromatography (Agilent 7890B GC, Agilent Technologies, Inc., Nanjing, China), equipped with NUKOLTM capillary column (30 m × 0.25 mm × 0.25 µm film thickness)^26^. The concentration of microbial protein (MCP) was measured using the Coomassie brilliant blue G250 staining method. Serum biochemical parameters, including glucose (GLU), triglyceride (TG), total protein (TP), albumin (ALB), Globulin (GLB) and Urea nitrogen (BUN) concentrations were measured using automatic biochemical analyzer (BS-420, Mindray Medical International Ltd., Shenzhen, China). The ammonia-N concentration of rumen content and feces was measured using the indigophenol blue colorimetric method with a 722N spectrophotometer (Shanghai PRECISION Scientific Instrument Co., Ltd., Shanghai, China) at a 700 nm wavelength.

### DNA extraction

The frozen samples were fully thawed, mixed well, and 0.3 g samples were taken for DNA extraction. DNA purification was performed using the QIAamp DNA stool Mini Kit (QIAGEN, Hilden, Germany). Subsequently, the concentration and purity of the DNA were assessed using a NanoDrop spectrophotometer (Nyxor Biotech, Paris, France) to ensure it met the required standards. The DNA samples were then amplified by PCR and subjected to 16 s rRNA high throughput sequencing.

### 16S rRNA sequencing and bioinformatics analysis

The V3-V4 regions of the bacterial 16S rRNA encoding sequences in rumen content and feces samples were amplified using primers 338F (5´-barcode-ACTCCTRCGGGAGGCAGCAG-3´) and 806R (5´-GGACTACCVGGGTATCTAAT-3´)^39^. The PCR amplified condition is as follows: 95 °C (2 min); 95 °C (30 s with 25 cycles); 55 °C (30 s); 72 °C (30 s); 72 °C (5 min). The quality of PCR products was assessed using a 1.5% agarose gel and purified using the AxyPrep DNA Gel Extraction Kit (Axygen Biosciences, Union City, CA, United States).

Sequencing was conducted on the Illumina MiSeq platform, and the obtained sequencing reads utilized the FLASH (version 1.2.7) and the QIIME (version 1.8.0) to consolidate the pair reads and analyze the data^40,41^ The high-quality reads were selected according to the criteria listed below: (a) minimum sequence length of 200 bp; (b) do not contain equivocal “N” bases; (c) average base quality score is not less than 25^42^. The duplicates in sequences that meet the criteria are removed, and the DADA2 algorithm (QIIME 2) was conducted to identify indel-mutations and substitutions^43^. The paired reads were trimmed and filtered, and no more than two expected errors per read (maxEE = 2). Paired reads were merged and chimera filtered. The RDP Classifier (http://rdp.cme.msu.edu/) analyzes the phylogenetic affiliation of each 16S rRNA gene sequence (herein called ASVs) through the silva (SSU132)16S rRNA database with a confidence threshold of 70%^44^. Rarefaction curves and diversity parameters (Chao1, Shannon, and Simpson) were used to assess sequencing depth and microbial structure (richness and diversity), respectively^45^, and the potential metabolic functions were prognosticated using PICRUSt (http://picrust.github.io/picrust/). Functional classification was performed using the Kyoto Encyclopedia of Genes and Genomes (KEGG) database.

### Statistical analysis

All statistical analysis were conducted using SPSS 23.0 (SPSS v. 20.0, SPSS Inc., Chicago, IL, United States). The data on fermentation parameters and serum biochemical parameters between two groups were analyzed using independent sample t-test, and the microbial data (such as microbial phylum, genus and functions) that identified as non-parametric data were analyzed using Wilcoxon-Mann–Whitney U test. The Bray–Curtis distance based principal coordinate analysis (PCoA) and the permutation multivariate analysis (PERMANOVA) were applied to evaluate whether the discrepant difference occurred in the microbial richness between groups. The linear discriminant analysis effect size (LEfSe) was employed to identify ASVs and metabolic pathways (at KEGG level 3) that were significantly enriched in two groups. The *P* < 0.05 was deemed to be the criterion for significant divergence. The heatmap plot was generated using R software (v.4.2.2) package “pheatmap” (v.1.0.12)^46^ through Hiplot Pro (https://hiplot.com.cn/), a comprehensive web service for biomedical data analysis and visualization.

### Supplementary Information

Supplementary Tables.

## Data Availability

In the present study, the 16S rRNA sequencing data of rumen and fecal microbiota had been submitted to National Center for Biotechnology Information Sequence Read Archive under the accession number PRJNA967832.
