# Supplementary material for: Differential responses of rumen and fecal fermentation and microbiota of Liaoning cashmere goats after 2-hydroxy-4-(methylthio) butanoic acid isopropyl ester supplementation
Source: Sci Rep. 2024 Apr 12;14:8505. doi: 10.1038/s41598-024-58581-y (PMC11009298; doi:10.1038/s41598-024-58581-y)
Supplement: Supplementary file 1 — Supplementary Tables. [file 41598_2024_58581_MOESM1_ESM.pdf]

Supplementary material prepared for Scientific Reports

Differential responses of rumen and fecal fermentation and microbiota of Liaoning cashmere goats after 2-hydroxy-4-(methylthio) butanoic acid isopropyl ester supplementation

Zhiqiang Zhong[1], **Peiyuan Sun**[1], **Yuning Zhang**[1], **Lingyun Li**[1], **Di Han**[2], **Xiaoguang Pan**[3], **Ruiyang Zhang**[1, 4*]

[1]College of Animal Science and Veterinary Medicine, Shenyang Agricultural University, Shenyang 110866, China.

[2]Liaoning Province Modern Agricultural Production Base and Construction Engineering Center, Shenyang 110032, China.

[3]School of Artificial Intelligence and Software, Liaoning Petrochemical University, Fushun 113001, China.

[4]State Key Laboratory of Swine and Poultry Breeding Industry.

*** Corresponding.** zhangruiyang@syau.edu.cn

Ingredient	(% of DM)	Nutrient composition	
Alfafa	30	ME (MJ/Kg)	8.77
Peanut vine	40	CP (%)	12.56
Corn	18	NDF (%)	43.69
Soybean meal	5.4	ADF (%)	28.90
Wheat bran	5	Ca (%)	0.99
Salt	0.6	P (%)	0.33
Dicalcium phosphate	0.5		
Premix	0.5		
Total	100		

The metabolic energy was calculated according to the NRC (2007).

Table S1 The comprehensive composition and nutrition of the basic diet in the present study.

Items	Group		SEM	*P* value
	CON	HMBi		
BW (kg)	43.64	44.13	1.512	0.881
ADG (g)	89.72	101.72	7.633	0.525
F/G	13.77	11.99	0.414	0.060

Table S2 Effects of 2-hydroxy-4-(methylthio) butanoic acid isopropyl ester (HMBi) supplementation on growth performance of Liaoning cashmere goats.

Items	Group		SEM	*P* value
	CON	HMBi		
Glucose, mmol/L	3.16	3.23	0.107	0.778
Triglyceride, mmol/L	0.30	0.27	0.022	0.633
Total protein, g/L	52.66	61.12	1.968	0.026
Albumin, g/L	19.17	22.07	0.992	0.149
Globulin, g/L	33.49	39.05	1.798	0.126
Urea nitrogen, mmol/L	5.61	6.17	0.188	0.138

Table S3 Effects of 2-hydroxy-4-(methylthio) butanoic acid isopropyl ester (HMBi) supplementation on serum biochemical indicators of Liaoning cashmere goats.
